# Identifying the predictors of turnover intention based on nurse managers’ toxic leadership behaviors among nurses in Iran: a cross-sectional correlational study

**DOI:** 10.1186/s12913-023-10046-0

**Published:** 2023-11-03

**Authors:** Elham Ahangari Nonehkaran, Naser Mozaffari, Sohrab Iranpour, Aghil Habibi Soola

**Affiliations:** 1 Department of Nursing, School of Nursing and Midwifery, Ardabil University of Medical Sciences, Ardabil, Iran; 2 Social Determinants of Health Research Center, Ardabil University of Medical Sciences, Ardabil, Iran

**Keywords:** Toxic leadership, Turnover intention, Nurse manager

## Abstract

**Background:**

Unfavorable leadership practices are a growing concern in the field of healthcare and nursing that have adverse consequences on nurses’ career outcomes. One of the undesirable leadership styles is the toxic leadership style. Considering the importance of nursing managers’ leadership style and its impact on nurses’ performance and the scarcity of studies in this field, the present study was conducted to determine toxic leadership behaviors in nursing managers and their relationship with the turnover intention among nurses.

**Methods:**

A multicenter cross-sectional correlational study. This study involved 551 nurses from 5 educational-medical centers in Ardabil province, north-western Iran. Three self-report scales, including The demographic and occupational information form, the Toxic Leadership Behaviors of Nurse Managers Scale (ToxBH-NM), and the turnover intention questionnaire were adopted for assessment purposes in this study. Data were analyzed using SPSS (Version 22) software using descriptive statistics, Pearson correlation coefficient test, t-test, ANOVA test, and multiple linear regression analysis.

**Results:**

Overall, 551 nurses participated in this research. There was a significantly positive relationship between Turnover intention and Toxic leadership behavior Subscales (r = 0.475, p < 0.001). Furthermore, multiple linear regression analysis showed Toxic leadership behavior Subscales (Intemperate behavior, Narcissistic behavior, Self-promoting behavior, and Humiliating behavior), Hospital, unit, Age, and Marital status predict Turnover intention when other variables are controlled.

**Conclusion:**

A leader who directly and indirectly adopts toxic behavior toward their employees destructively affects both individuals and organizations. Nurses who work for a manager exhibiting toxic leadership behaviors demonstrated higher turnover intention.

## Background

Nursing leaders today are faced with many complex issues that are increasingly caused by the healthcare system. Increasing healthcare costs, continuous financial restrictions and budget cuts, increasing patient awareness, and shortage of nursing personnel require the participation of nursing leaders to direct the organization to ensure and provide healthcare services while achieving the desired goals [1, 2]. Good leadership practices that rely on criteria such as respect, trust, and open communication are necessary not only in providing safe and high-quality care but also in creating a quality work environment where nurses are valued and respected, aiming to increase work motivation, job satisfaction, and organizational commitment in nurses [3]. There is a significant positive relationship between desirable leadership and increased job satisfaction, as well as a negative relationship between desirable leadership and inappropriate employee behaviors at work and turnover intention [4].

However, unfavorable leadership practices are a growing concern in the field of healthcare and nursing that have adverse consequences on nurses’ career outcomes [5, 6]. One of the undesirable leadership styles is the narcissistic leadership style. In this type of leadership style, the leader shows positive self-assertion, which is associated with poor job outcomes such as job dissatisfaction and reduced interest in the job [7]. Nurses who are under the supervision of a leader with a laissez-fair leadership style often have high stress, low job satisfaction, and less desire to stay in their organization [8]. Yet, the toxic leadership style is another type of undesirable leadership style that is being considered increasingly in management-related studies and has attracted many researchers in the last few years [9]. This type of leadership style has a negative and highly significant relationship with organizational performance [10]. As a group of behaviors or practices that are destructive in nature and directly or indirectly harm employees and the organization [11], toxic leadership behaviors can be divided into four Subscales of intemperate, narcissistic, self-promoting, and humiliating behaviors [5].

A review of nursing and healthcare literature shows that unfavorable leadership styles such as abusive leadership, narcissistic leadership, and laissez-faire leadership are strongly associated with negative outcomes among nurses and patients, as nurses who work under unfavorable leadership have low job satisfaction [11–13] and a higher tendency to turnover intention [14–16]. Also, favorable leadership styles such as transformational leadership, authentic leadership, and transactional leadership are negatively associated with turnover intention among nurses [8, 17]. The results of the study conducted in Iran showed Nursing Manager’s leadership style was transactional leadership. Both transformational and transactional leadership style have a significant relationship with job stress and anticipated staff turnover. A positive relationship was found between a laissez-faire leadership style with job stress and anticipated turnover [8].

One of the effective factors in employees’ turnover intention is the leadership style [12]. The findings show that nurses who work with a transformational leader have higher job satisfaction and less turnover intention, and nurses who work under a manager with a toxic leadership style have lower job satisfaction, higher stress, and frequent absenteeism [11]. The average rate of turnover intention varies from country to country, such as Indonesia [11] and South Korea [13] among Asian countries with the highest rates of 15% and 25%, respectively, and the United States among western countries with 18% [14]. In Iran [15] and in Ardabil city, the average turnover intention among nurses during the outbreak of the Covid-19 disease was 32.7%. The loss of experienced nurses has a negative impact on the provision and continuity of patient care services and may lead to more side effects, loss of nursing care, and patient mortality [16].

Although no specific theory is available to explain the occurrence of toxic leadership, previous studies show that the presence of several factors in an organization can facilitate the occurrence of toxic leadership [17], Toxic leadership appears as a result of the relationship between a sensitive subordinate, a destructive leader, and an intemperate organization [18]. To date, studies in this field have been mainly limited to the financial and academic sectors [19, 20], and only a few studies have been conducted in the field of nursing. These studies report a significant decrease in work productivity and employment, the emergence of physical and emotional distress, and turnover intention among nurses who have experienced toxic leadership [6]. Therefore, considering the importance of nursing managers’ leadership style and its impact on nurses’ performance and the scarcity of studies in this field, the present study was conducted to determine toxic leadership behaviors in nursing managers and their relationship with the turnover intention among nurses.

## Methods

### Study design

This multicenter cross-sectional correlational study was performed from August to September 2022 in Ardabil province, north-western Iran.

### Participants and setting

The statistical population of this study consisted of nurses, who were selected from three units (general, emergency, and intensive) in five educational-medical centers affiliated with Ardabil University of Medical Sciences (General centers, Nephrology and Ophthalmology centers, Trauma centers, Gynecology centers, and Neonatology centers) To achieve an 80% power, with alpha set at 0.05 and small effect size set at 0.03, the desired sample size was 551 nurses, as determined using the G*power program. The sampling process included stratified random sampling in which each hospital was considered as a stratum and simple random sampling was used to select samples from each hospital. Considering the number or proportion of nurses of each hospital among all the nurses of the Ardabil city, the proportion for each hospital was considered. The inclusion criteria of the study consisted of having working experience in present unit for a minimum of 6 months and being active during the data collection stage. All those who were on leave during the study time and had incomplete questionnaires were excluded from the research. In this study, nurses who hold titles such as Head nurse, Supervisor and Matron were considered to be nurse managers. Initially, the researchers obtained a permit from the Ethics Committee of the University of Medical Sciences and received a letter of recommendation from the Vice Chancellor for Research. The letter was presented to the officials of educational hospitals in Ardabil. The researchers were introduced to the nurses by the nursing offices of the mentioned hospitals. Before sampling, a short introduction of the study design and purpose was presented to the prospective participants. The paper version of the questionnaire was then distributed among the participants during working hours. Written informed consent was obtained from the participants in this study. The completed questionnaires were delivered to the researcher in sealed envelopes.

### Data collection

Three self-report scales, including The demographic and occupational information form, the Toxic Leadership Behaviors of Nurse Managers Scale (ToxBH-NM), and the turnover intention questionnaire were adopted for assessment purposes in this study.

### Demographic and occupational information form

demographic and occupational information form included age, Year in the present unit, Year in nursing profession, gender, marital status, education, job role, unit and hospital.

### Toxic leadership behaviors of nurse managers scale (ToxBH-NM)

ToxBH-NM is a 30-item instrument that was developed by Labrague, Lorica et al. in 2020 [21]. The questionnaire consists of four Subscales. Including intemperate (n = 15 items), narcissistic (n = 9 items), self-promoting (n = 3 items) and humiliating (n = 3 items) behaviors. The intemperate behaviors Subscale pertains to aggressive actions or behaviors by NMs, which reflect a lack of emotional intelligence. The narcissistic behaviors Subscale pertains to actions or behaviors shown by NMs that are motivated by self-interest. The self-promoting behaviors Subscale pertains to behaviors or actions by NMs to help advance their own personal while neglecting the employees’ or organization’s welfare. Lastly, the humiliating behaviors Subscale reflects actions or behaviors shown of NMs that could cause embarrassment to their employees. Each item of the scale was rated on a five-point Likert scale (1/not at all to 5/frequently) and the sum of the scores varies between 30 and 150 with higher scores indicating higher Toxic Leadership. The Cronbach’s alpha value in the study conducted by Labrague, Lorica et al. was 0.975 [21]. After obtaining permission from the original designer, the English version of this questionnaire was translated into Persian by an independent translator. To determine the content validity index and ratio, a questionnaire was given to 12 faculty members of Ardabil University of Medical Sciences (Faculty of Nursing and Midwifery). The Content Validity Index (CVI) was evaluated separately by experts using three criteria of simplicity, appropriateness, and certainty based on a four-part spectrum (for example, in terms of simplicity, quite simple, somewhat complex, and complex) for each question. Relevant ratings were given. Finally, the content validity index was 0.90. Moreover, the test-retest method was used to assess the reliability of the questionnaire. Thirty questionnaires were distributed among the nurses at the same time interval, then the agreement of the answers was evaluated, and a coefficient of *r* = 0.96 was obtained, which indicates the compatibility of the questionnaire. The researcher confirmed that participants understood each item in the questionnaire during the pilot study; therefore, there were no changes in the items of the questionnaire. In the main study, Cronbach’s alpha ranged from 0.89 to 0.96 for each subscale [21]. In the current study, Cronbach’s alpha for each subscale ranged from 0.80 to 0.93, and it was 0.96 for the whole questionnaire.

### Turnover intention questionnaire

Turnover Intention in nurses were measured using the Kim et al.‘s Turnover Intention Questionnaire [22]. This questionnaire includes 15 questions scored on a five-point Likert scale (1 = strongly disagree to 5 = strongly agree), and the sum of the scores varies between 15 and 75 with higher scores indicating higher turnover intention. The Persian version of the questionnaire has been validated in Iran and its Cronbach’s alpha was 0.76 [23]. The Cronbach’s alpha value of the Turnover Intention Questionnaire in this study was 0.89.

### Ethical considerations

After obtaining approval for the research plan and a license to conduct research from the Research Ethics Committee of Ardabil University of Medical Sciences (IR.ARUMS.REC.1401.084), and coordinating with the head of the hospital, security guards, and ward nurses, the researcher introduced herself to the research samples and after explaining the purpose of the research, obtained their written consent to participate in the research. All research samples were assured that the information obtained was confidential and did not need to be named.

### Data analysis

After collecting the questionnaires, the data were analysed using SPSS-ver. 22. Kolmogorov-Smirnov test was used to evaluate the normality of the data. The results of the normality test showed that the studied data were normal. At first, data related to demographic and occupational characteristics were reported using percentages, means, and standard deviations. Second, the independent t-test was used to investigate the relationship between Turnover intention and Gender, marital status, Education, and Job role. Further, the ANOVA test was used to investigate the relationship between Turnover intention and unit and Hospital. Pearson correlation analysis was used to investigate the relationship between Turnover intention and Age, Year in the present unit and Year in nursing profession. Pearson correlation analysis was used to investigate the relationship between Turnover intention and Toxic leadership behavior Subscales. Factors affecting Turnover intention were identified as predictors via multiple linear regression analysis.

## Results

Overall, 551 nurses participated in this research. The mean ± SD scores of participants’ age, Year in the present unit, and Year in nursing profession were 32.44 ± 5.82years, 5.91 ± 4.73, and 8.78 ± 5.41, respectively. The majority of participants were Female (81.7%), had a bachelor’s degree (94.7%), were married (61.9%), were Nurse (96.4%), were General unit (46.5%), and 53.2%of nurses were working in General hospital. The demographic characteristics of study participants and statistical analyses are shown in Table 1. The results of the t-test and ANOVA showed a significant relationship between Turnover intention and years Gender (t=-2.15, p = 0.032), Marital status (t=-0.845, p = 0.399), Education (t = 0.472, p = 0.637), Job role (t=-0.593, p = 0.553), unit (f = 2.706, p = 0.068), Hospital (f = 4.692, p = 0.001) (Table 1).

**Table 1:** Descriptive statistics of participants and their relationship with the total score of Turnover intention (n = 551)

Characteristics	Categories	Mean	SD	P value
Age		32.44	5.82	r = -0.11
				p = 0.009
Year in the present unit		5.91	4.73	r = -0.040
				p = 0.349
Year in nursing profession		8.78	5.41	r = -0.110
				p = 0.010
		**N**	**%**	
Year in nursing profession group	< 1	37	6.7	F = 1.678
	2–5 Years	164	29.8	P = 0.154
	6–10 Years	155	28.1	
	11–15 Years	125	22.7	
	> 15	70	12.7	
Gender	Male	101	18.3	t = -2.150
	Female	450	81.7	p = 0.032
Marital status	Married	341	61.9	t = -0.845
	Single	210	38.1	p = 0.399
Education	Bachelor	522	94.7	t = 0.472
	Master’degree	29	5.3	p = 0.637
Job role	Nurse	531	96.4	t = -0.593
	Department Secretary	20	3.6	p = 0.553
unit	Emergency	177	32.1	F = 2.706
	Intensive	118	21.4	P = 0.068
	General	256	46.5	
Hospital	General	293	53.2	F = 4.692
	Nephrology and Ophthalmology	40	7.3	P = 0.001
	Trauma	92	16.7	
	Gynecology	62	11.3	
	Neonatology	64	11.6	

The mean ± SD score of Toxic leadership behavior was 2.208 ± 0.858. the mean ± SD scores of Toxic leadership behavior Subscales (Intemperate behavior, Narcissistic behavior, Self-promoting behavior, and Humiliating behavior) were reported to be 2.100 ± 0.862, 2.305 ± 0.965, 2.387 ± 1.142, and 2.278 ± 1.045 respectively (Table 2). Pearson correlation test was used to test the relationship among Turnover intention, Toxic leadership behavior and Toxic leadership behavior Subscales (Intemperate behavior, Narcissistic behavior, Self-promoting behavior, and Humiliating behavior). There was a significantly positive relationship between Turnover intention and Toxic leadership behavior Subscales (r = 0.475, p = 000, N = 551) (Table 2)

**Table 2:** Descriptive statistics and Correlations among the study variables (n = 551)

Variable	Mean(SD)	Range	Turnover intention r(p)
Total Toxic leadership behavior	2.208(0.858)	1–5	0.475(< 0.001)
Intemperate behavior	2.100 (0.862)	1–5	0.470(< 0.001)
Narcissistic behavior	2.305(0.965)	1–5	0.449(< 0.001)
Self-promoting behavior	2.387(1.142)	1–5	0.351(< 0.001)
Humiliating behavior	2.278(1.045)	1–5	0.339(< 0.001)
Turnover intention	2.705(0.802)	1–5	1

Multiple linear regression predicts factors that affect Turnover intention and toxic leadership behaviour nurse managers. Toxic leadership behavior Subscales (Intemperate behavior, Narcissistic behavior, Self-promoting behavior, and Humiliating behavior), Hospital, unit, Age, and Marital status predict Turnover intention when other variables are controlled. all subscales of Toxic leadership behavior, was significantly and positively associated with the Turnover intention in the crude model. Model 1 after adjusting for Toxic leadership behavior Subscales, Hospital, unit, Age, and Marital status was significantly and positively associated with the Turnover intention like crude model. While the crude model and model 1 found a significant association between all Subscales toxic leadership behaviour and Turnover intention, models 2 only showed significant association between the two Subscales toxic leadership behaviour (Intemperate behavior, Narcissistic behavior) and Turnover intention. (Table 3)

**Table 3:** Regression analyses of the association between Toxic leadership behavior and Turnover intention

Variables	Model	Beta	Sig	Adjusted R Square	Lower Bound	Upper Bound	
Intemperate behavior	Crude	0.470	< 0.001	0.219	0.368	0.506	
	Model 1	0.529	< 0.001	0.268	0.413	0.579	
	Model 2	0.356	< 0.001	0.280	0.207	0.450	
Model 1: Adjusted for Intemperate behavior, Hospital, unit, Age, Marital status Model 2: Adjusted for model 1 plus Narcissistic behavior, Self-promoting behavior, Humiliating behavior							
Narcissistic behavior	Crude	0.449	< 0.001	0.200	0.311	0.435	
	Model 1	0.507	< 0.001	0.247	0.348	0.489	
	Model 2	0.209	0.010	0.280	0.062	0.292	
Model 1: Adjusted for Narcissistic behavior, Hospital, unit, Age, Marital status Model 2: Adjusted for model 1 plus Intemperate behavior, Self-promoting behavior, Humiliating behavior							
Self-promoting behavior	Crude	0.351	< 0.001	0.122	0.192	0.302	
	Model 1	0.416	< 0.001	0.162	0.232	0.355	
	Model 2	0.054	0.412	0.280	-0.056	0.128	
Model 1: Adjusted for Self-promoting behavior, Hospital, unit, Age, Marital status Model 2: Adjusted for model 1 plus Intemperate behavior, Narcissistic behavior, Humiliating behavior							
Humiliating behavior	Crude	0.339	< 0.001	0.114	0.200	0.321	
	Model 1	0.352	< 0.001	0.134	0.204	0.338	
	Model 2	-0.048	0.396	0.280	-0.111	0.047	
Model 1: Adjusted for Humiliating behavior, Hospital, unit, Age, Marital status Model 2: Adjusted for model 1 plus Intemperate behavior, Narcissistic behavior, Self-promoting behavior							

Dependent variable: Turnover intention

## Discussion

The shortage of experienced nurses is a big concern in health organizations. Considering the increasing rate of nurses leaving their job, it is necessary to find out why nurses leave healthcare organizations because nurses are considered the most valuable asset in healthcare systems [24]. Managerial support is an important predictor of turnover intention [25], and the leadership styles of nursing managers are thought to determine the nurses’ turnover intention [26]. Considering the lack of available evidence on how toxic leadership affects nurses’ work outcomes (e.g., work contentment, job commitment and work engagement) [17]) and the increasing number of nurses who are willing to leave their organizations and jobs, the present study aimed to determine the predictors of turnover intention based on toxic leadership behaviors of nursing managers in the nurses of Ardabil province.

In the present study, the mean turnover intention among 551 nurses was 2.70 (score range 1–5). The results of the study conducted by Hariri showed that turnover intention among nurses in Tehran was 3.35 (score range 1–5) [27]. The mean turnover intention during the outbreak of Covid-19 in Ardabil was 41.73 (score range 15–75) [23]. Liou et al. reported that turnover intention among Asian nurses in the U.S. was 2.45 (score range 1–5) [28]. Cai et al. also conducted a study in China and found that the mean turnover intention was 3.39 (score range 1–5) [29]. Evidence suggests that the average rate of turnover intention among nurses varies in different societies, which may be attributed to multiple definitions of the phenomenon of turnover intention or differences in research environments in the mentioned studies.

The results of the study showed a positive and significant relationship between turnover intention and toxic leadership behaviors of nursing managers, as with a rise in the intensity of toxic leadership behaviors in nursing managers, turnover intention increased in nurses. This result is consistent with the finding of Labrague et al. Nurses who work under the supervision of a manager with toxic leadership behaviors have less job satisfaction, higher stress, frequent absences, and a higher turnover intention [11]. Studies conducted in other organizations have identified a strong relationship between toxic leadership behaviors and poor job performance, low work motivation, frequent delays and absences, low employee productivity, and an increased turnover intention [9, 30]. It is worth noting that the mean scores of all dimensions of toxic leadership behaviors were below average. The results of the study conducted by Labrague et al. also showed the average scores of all dimensions of toxic leadership behaviors were low or mild [11]. The results of the study conducted by Shipl et al. indicated a slightly moderate mean percentage score for toxic leadership [31]. Abou-Ramadan et al. reported that more than one-third of nursing staff perceived that their leaders had a high and moderate level of narcissism, and about one third had high and moderate unpredictability behaviors of toxic leadership [32]. In current study, among the four subscales of toxic leadership behaviors of nursing managers (intemperate, narcissistic, self-promoting, and humiliating behaviors), narcissistic behaviors have scored the maximum. The results of the study conducted in Ghana indicated nurses appraised the leadership behaviour of nurse managers to be toxic, with most managers exhibiting narcissistic leadership behaviour [33]. Farghaly et al. reported that toxic leadership behavior is above average [10]. These differences are probably due to cultural differences or the way nursing managers are trained. It can also be argued that nursing managers who have previously worked as nurses in hospitals show less toxic behaviors after being employed as a manager.

The results of the present study showed a significant and negative relationship between the turnover intention and age, as with an increase in age, the tendency to leave the job decreased. The results of Liou’s study [28] on Asian nurses in America and Hart’s study [34] on American nurses showed a significantly negative relationship between age and turnover intention. These findings may be due to the fact that younger nurses experience high stress and anxiety due to their low experience in performing bedside tasks and communicating with colleagues and even poor decision-making ability, and on the contrary, nurses with more work experience feel comfortable in their job due to the ability to make decisions and communicate properly.

The present study showed that married nurses had less turnover intention. Hariri showed that the average turnover intention was lower among married nurses [27]. The results of the study conducted by Mirzaei et al. on the relationship between marital status and turnover intention are consistent with the results of the present study [23].

The results of the current study indicated the highest turnover intention in the hospital and general departments. The studies conducted by Hariri and Mirzaei showed that the highest turnover intention was related to the general wards [23, 27]. Due to the large number of beds in general wards, high workload, lack of manpower, and non-compliance with the standards of nurse-patient ratio, the turnover intention in these departments is higher.

### Limitation

There are some limitations in this research that may have affected the results and should be considered when interpreting the findings. First, the information related to the variables of this study was collected through self-report not through accurate information collection strategies such as actual observations. Second, special nursing requirements such as fatigue, work pressure, and time limit affected the completion of the questionnaires. To solve these two problems, we tried to get the cooperation of nurses as much as possible by stating the objectives of the study and its benefits in order to encourage them to answer the questions accurately and reduce the possibility of bias. Finally, the study sample only included nurses working in the teaching hospitals of Ardabil city. Therefore, the results should be generalized with caution.

## Conclusion

The present study showed a positive and significant relationship between nursing managers’ toxic leadership style and nurses’ turnover intention. The issue of toxic leadership style has been the focus of researchers in the field of nursing in recent years because a leader who directly and indirectly adopts this toxic behavior towards their employees destructively affects both individuals and organizations. As a result, it is highly essential that nursing managers apply appropriate training strategies to reduce the severity of toxic leadership styles and replace them with suitable and supportive leadership styles in order to reduce their destructive effects. Organizational measures are vital to address toxic leadership behaviours among nurse managers, which could potentially pose a threat to patients and nurses. Nurse managers, particularly the less experienced, may benefit from mentoring from experienced nurse managers. It is suggested that Training sessions for stress management, coping skills, and resilience should be implemented by the hospital administrators to help nurse managers to handle their emotions in a healthy technique so as to effectively avoid showing toxic behaviours. Further studies are needed to assess environmental factors and their relationship with toxic leadership behaviors among nursing managers.

## Data Availability

The datasets used and/or analysed during the current study available from the corresponding author on reasonable request.
